# Dietary Nitrosamines from Processed Meat Intake as Drivers of the Fecal Excretion of Nitrosocompounds

**DOI:** 10.1021/acs.jafc.4c05751

**Published:** 2024-07-29

**Authors:** Sergio Ruiz-Saavedra, Tuulia Kreetta Pietilä, Aida Zapico, Clara G. de los Reyes-Gavilán, Anne-Maria Pajari, Sonia González

**Affiliations:** † Department of Microbiology and Biochemistry of Dairy Products, Instituto de Productos Lácteos de Asturias (IPLA-CSIC), 33300 Villaviciosa, Spain; ‡ Department of Food and Nutrition, University of Helsinki, 00014 Helsinki, Finland; § Department of Functional Biology, University of Oviedo, 33006 Oviedo, Spain; ∥ Diet, Microbiota and Health Group, Instituto de Investigación Sanitaria del Principado de Asturias (ISPA), 33011 Oviedo, Spain

**Keywords:** food processing, nitrosamines, N-nitroso compounds, intestinal mucosa lesions, hyperplastic polyps, conventional adenomas, fecal mutagenicity

## Abstract

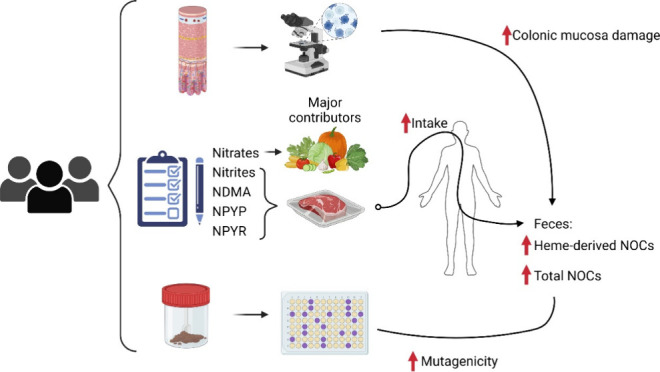

Diet is one of the main exogenous sources of potentially carcinogenic nitrosamines (NAs) along with tobacco and cosmetics. Several factors can affect endogenous N-nitroso compounds (NOCs) formation and therefore the potential damage of the intestinal mucosa at initial colorectal cancer stages. To address this issue, 49 volunteers were recruited and classified according to histopathological analyses. Lifestyle and dietary information were registered after colonoscopy. The mutagenicity of fecal supernatants was assayed by a modified Ames test. Fecal heme-derived NOCs and total NOC concentrations were determined by selective denitrosation and chemiluminescence-based detection. Results revealed processed meats as the main source of dietary nitrites and NAs, identifying some of them as predictors of the fecal concentration of heme-derived and total NOCs. Furthermore, increased fecal NOC concentrations were found as the severity of colonic mucosal damage increased from the control to the adenocarcinoma group, these concentrations being strongly correlated with the intake of the NAs N-nitrosodimethylamine, N-nitrosopiperidine, and N-nitrosopyrrolidine. Higher fecal NOC concentrations were also noted in higher fecal mutagenicity samples. These results could contribute to a better understanding of the importance of modulating dietary derived xenobiotics as related with their impact on the intestinal environment and colonic mucosa damage.

## Introduction

The consumption of red and processed meat has been assessed by the World Cancer Research Fund (WCRF) as a risk factor for colorectal cancer (CRC) in humans.^1^ The presence of heme iron, the thermal formation of different carcinogens during cooking, the generation of lipid and protein oxidation products, and the formation of exogenous N-nitroso compounds (NOCs) in cured meats or endogenous NOCs in the gastrointestinal tract are considered among the possible mechanisms underlying this association.^1^ Exogenous NOCs mainly comprised nitrosamides and nitrosamines (NAs). Nitrosamides are chemically unstable and eventually decompose.^2^ Therefore, the most abundant NOCs found in foods are NAs such as N-nitrosodimethylamine (NDMA), N-nitrosopiperidine (NPIP), and N-nitrosopyrrolidine (NPYR).^3^ In this regard, the European Food Safety Authority has recently issued a technical report on the risks to human health associated with the intake of NAs in foods, concluding that exposure to a panel of 10 carcinogenic NAs raises a health concern.^4^ Although unprocessed and uncooked meat may contain traces of NAs, these compounds have been detected at higher concentrations in cured meats, smoked fish, cheese, preserved vegetables, beer, and human milk, highlighting the importance of cooking and processing methods in the final concentrations.^2^ Other nondietary major exposure sources to NAs include tobacco and personal care products.^4^ Previous studies in animal and cell models have proposed that some NAs may be linked to a higher risk of CRC through the increase of fecal genotoxicity and the formation of highly reactive diazonium ions, which can generate DNA adducts.^5−7^ Furthermore, the excess of protein fermentation in the intestine is associated with higher levels of amines and other compounds potentially toxic to the gut mucosa such as heme iron, which induces the generation of free radicals in the colon.^8,9^ Most of the available research in humans comes from epidemiological studies in which the assessment of NA exposure is limited. The food frequency questionnaire (FFQ), a commonly employed method for intake assessment, estimates long-term dietary intake and enables the comparison among individuals in a population.^10^ However, it lacks precision and the ability to reflect the exposure to other environmental sources of these compounds such as tobacco, water, or cosmetic products.^11^ Moreover, predicted endogenous concentrations of NOCs have been calculated to be 100 times higher than the estimated dietary intakes.^12^ In the gastrointestinal tract, dietary components such as red meat, protein, and NOCs precursors such as nitrates, nitrites, and heme iron can contribute to further nitrosation processes and therefore to endogenous formation of nitrosyl heme (heme NOCs), S-nitrosothiols (SNOs) and NAs by acid-catalyzed, intestinal cell or microbiota mediated pathways, finally being excreted in feces as total (apparent) NOCs.^12−16^ However, there are no studies that have analyzed the correlation between the consumption of these dietary sources and the fecal NOC concentrations obtained by analytical determinations in the context of CRC. Based on this evidence, a more comprehensive approach is needed to lay the groundwork for a better understanding of the complex associations between diet and cancer in the long term.

In the present study, we therefore investigated the impact of diet and dietary xenobiotics on fecal NOC concentrations and their association with fecal mutagenicity in a sample population of adults at different stages of intestinal mucosa damage in the progression to CRC, after biopsy examination.

## Materials and Methods

### Study Design and Volunteers

This study is part of broader projects related to the effect of diet and dietary xenobiotics on intestinal mucosa and related gut microbiota profiles in the context of CRC (MIXED and MiToxicDiet projects). The recruitment of volunteers was carried out from October 2019 to December 2021 by Facultatives of the Digestive Service from the Central University Hospital of Asturias (HUCA) and the Carmen and Severo Ochoa Hospital in Cangas de Narcea, Asturias, Spain. Volunteers were selected among individuals enrolled in a colon cancer screening program. Inclusion criteria were being between 40 and 79 years old and not referring to the intake of omeprazole, antibiotics, corticoids, or nonsteroidal anti-inflammatory drugs. In addition, having specific cancer treatment at the time of the study or in the previous 2 months, previous surgery of the digestive system, autoimmunity, altered thyroid function, or history of diabetes or goiter were exclusion criteria. Those individuals interested in participating were informed of the objectives of the study and signed an informed consent form. A total of 49 subjects were included in the study. Patients were asked to provide a stool sample collected prior to preparation for colonoscopy. During the procedure, a biopsy for the removal of tissue samples was performed. Biopsies were examined at the Department of Anatomical Pathology of HUCA, as described elsewhere.^17^ After biopsy examination patients were classified into four histopathology groups in order of increasing risk of CRC development: nonpathological control (NP) (*n* = 18), hyperplastic polyps (HP) (*n* = 10), conventional adenomas (CA) (*n* = 18), and adenocarcinomas (AC) (*n* = 3).

This project was evaluated and approved by the Regional Ethics Committee of Clinical Research of Asturias (ref. 163/19) and by the Committee on Bioethics of CSIC (ref. 174/2020). The procedures were performed in accordance with the fundamental principles set out in the Declaration of Helsinki, the Oviedo Bioethics Convention, and the Council of Europe Convention on Human Rights and Biomedicine, as well as in Spanish legislation on bioethics. Directive 95/46/EC of the European Parliament and the Council of October 1995, on the protection of individuals regarding the processing of personal data, was strictly followed.

### Nutritional Assessment

Dietary information was obtained from volunteers through a personalized interview conducted by a trained interviewer when they were informed about the colonoscopy results at medical consultation. Exceptionally, as a result of the COVID-19 restriction of visitors to hospitals in Spain during the pandemic, some of the surveys were conducted through online tools. For this purpose, a 155-item semiquantitative FFQ previously developed by the research group and validated for the estimation of dietary xenobiotics intake was used.^18^ In addition to food and culinary preparations, the specific type of food was recorded, as well as cooking and processing methods and other related questions such as the degree of doneness or temperature, when necessary. Information relative to dietary assessment for the estimation of the dietary intake of xenobiotics has been previously published.^17^ The classification of foods into food groups was carried out according to the Centre for Higher Education in Nutrition and Dietetics (CESNID) criteria.^19^ Then, food composition tables of CESNID were used to transform food consumption into energy, macro- and micronutrient intake.^19^ The content of nitrates, nitrites, and the exogenous NAs NDMA, NPIP, and NPYR was estimated using the European Prospective Investigation into Cancer and Nutrition (EPIC) Potential Carcinogen Database and other databases previously indicated.^17,20^ Specifically, NA concentration values were compiled for processed meats typically consumed in the geographical region, such as cured ham (pork meat), chorizo (a Spanish sausage of minced pork meat combined with paprika and spices), and blood sausage (made of cooked blood generally obtained from pork, pork fat, onion, and spices).^21,22^ Out of the 74 foods in the final database consisting of 258 different items regarding nitrates, nitrites, and NAs concentration values, 54 foods were consumed in the sample. Of these, 27 were vegetable products, and 10 were processed meats. The phenolic content of the foods was extracted from Phenol Explorer 3.6 and fiber content from the tables by Marlett and Cheung.^23,24^ Oxygen Radical Activity Capacity (ORAC) was calculated based on the article by Wu et al.^25^ For each dietary compound of interest, the food sources with at least 5% contribution in each case were considered. Heme iron intake was calculated by assuming that heme iron was 40% of total iron contained in all meats, fish, and eggs, as proposed by Monsen and Balintfy.^26^ Dietary information was obtained for 36 volunteers from NP (*n* = 11), HP (*n* = 8), CA (*n* = 14), and AC (*n* = 3) groups.

### General Characteristics

During personalized interviews, sleeping hours and physical activity were recorded as the average self-referred time per day for each individual considering the last year, while the number of depositions was recorded as the self-referred times during a normal week. Information on smoking habits was obtained by asking about cigarette smoking throughout life. The anthropometrical parameters height (m) and weight (kg) were taken by standardized protocols.^27^ Body mass index (BMI) was calculated using the formula weight/(height)^2^.

### Measurement of Fecal Total NOCs and Heme NOCs

Fecal homogenates were prepared by diluting samples (1:5) with ultrapure Milli-Q water (resistivity 18,2 MΩ cm at 25 °C, Millipore, Rios30) and homogenized with T-18 Digital Ultra Turrax (IKA, Germany). Total NOCs and heme NOCs were analyzed from the fecal homogenates using selective denitrosation and chemiluminescence-based detection by Ecomedics CLD 88 Exhalyzer (Eco Medics, Switzerland) equipped with a custom-made liquid purge vessel and a NaOH (1 mol/L, kept at 4 °C) trap as presented in previous studies.^28,29^ Unless otherwise stated, the chemicals were obtained from Sigma-Aldrich (Merck Life Science).

Preservation solution [250 mg of N-ethylmaleimide (*M* = 125 g/mol), and 78 mg of diethylenetriaminepentaacetic acid (*M* = 393 g/mol) in 20 mL of Milli-Q water] was used to prevent artificial nitrosation via binding of metal iron and alkylating free thiol groups, whereas acid sulphanilamide (SA) solution [5 g/100 mL in 1 M hydrogen chloride (HCl)] was used to remove nitrite.^30^ Use of selective denitrosation enables the indirect determination of heme NOCs. Therefore, mercury(II) and ferricyanide stable nitroso compounds (nonheme) were determined using aqueous HgCl_2_ (10 mM HgCl_2_, *M* = 271.5 g/mol) and K_3_Fe(CN)_6_ (10 mM K_3_Fe(CN)_6_, *M* = 368.35 g/mol).

Fecal homogenate (100 μL) was incubated at room temperature for 5 min with 100 μL preservation and 500 μL of SA solution with or without HgCl_2_ and K_3_Fe(CN)_6_ (100 μL/each). Fifty microliters were injected into the purge vessel containing 15 mL of reducing agent triiodine mixture [1 g potassium iodine (*M* = 166.0 g/mol) and 0.65 g iodine (*M* = 253.81 g/mol, Acros Organics, UK)], 70 mL glacial acetic acid (WWR Chemicals, France), 20 mL Milli-Q water, and Antifoam B Emulsion (#A6707) kept at 60 °C that releases nitrogen oxide (NO) from NAs, SNOs, alkyl nitrites, and iron nitrosyl compounds. System helium gas flow (110 mL/min, adjusted 0 ± 5.0 mbar) mixes the sample and transfers the released NO to the CLD 88 via a condenser, the chemical trap, and a 0.20 μm polypropylene filter. Each sample was injected twice, first for total NOC and then for heme NOC determination. 5–10 min were left in between the injections for the signal to return to baseline. The triiodine mixture was changed at intervals of three samples.

The signal was recorded with a PowerChrom 280 system and analyzed by the instrument software (PowerChrom, eDAQ, Australia). Cutoff frequency for the signal was set to 0.05 Hz to reduce the noise. Calibration curves of known standards (5 to 950 picomoles, pmol) of aqueous sodium nitrite (NaNO_2_) were used for quantification by comparing the area under the curve to the area of known standards. Calibration curve determination coefficient (*R*^2^) > 0.96 was accepted. Aliquots of a pooled sample were used as the internal control for monitoring interday reproductivity of the system and calibration curves prior to injecting samples. Peak selection was done based on visual evaluation and the determination of the quantification limit was set to 2.5 pmol. Heme NOCs were determined by subtracting the values of mercury(II) and ferricyanide stable compounds from the total NOC. Concentrations were obtained for 49 volunteers from NP (*n* = 18), HP (*n* = 10), CA (*n* = 18), and AC (*n* = 3) groups and expressed as pmol per milligram of fecal sample (pmol/mg).

### Fecal Mutagenicity

The Ames test assayed the mutagenicity of fecal supernatants without metabolic activation against the strain *Salmonella enterica* serovar Typhimurium TA100. The 5051 Muta-ChromoPlateTM kit (EBPI, Ontario, USA) was used. Filtered fecal supernatant dilutions were mixed with the bacteria grown over 16 h at 37 °C in the sterile medium provided by the manufacturer and the solution mix containing Davis-Mingoli salts, d-glucose, bromocresol purple, D-biotine, and l-histidine as indicated by the manufacturer. Positive control (including sodium azide as a mutagen, grown bacteria, and solution mix), negative control (including only solution mix), and the appropriate series of dilutions of fecal supernatants were added to 96-well microtiter plates containing 200 μL per well and incubated at 37 °C for 5 days. Reversion rates (RR) were calculated for conditions where less than 96 revertant wells per plate and more than 48 revertant wells in the positive control were obtained. Considering the dilution factor, the levels of mutagenicity were expressed as the mean of values corresponding to the three dilutions tested per sample and were arbitrarily classified as “low” [180–299] (*n* = 6), “medium” [300–599] (*n* = 27) or “high” [600–1474] (*n* = 8) mutagenicity.^17^ The interference with l-histidine during the mutagenicity assay was ruled out as indicated in Ruiz-Saavedra et al.^17^ Fecal mutagenicity values were obtained for 41 volunteers from NP (*n* = 15), HP (*n* = 9), CA (*n* = 14), and AC (*n* = 3) groups.

### Statistical Analyses

Results were analyzed using the IBM SPSS software version 25.0 (IBM SPSS, Inc., Chicago, IL, SA). GraphPad Prism 9, RStudio version 1.4.3, and BioRender software were used for graphical representations. Overall, categorical variables were summarized as numbers and percentages and continuous ones as mean and standard deviation. Fisher tests were performed for categorical variables (*p*-value <0.05). For continuous variables, the goodness of fit to a normal distribution was checked by means of the Kolmogorov–Smirnov test. When normality of variables was achieved, *T*-tests were performed; otherwise, Mann–Whitney *U* tests were applied (*p*-value <0.05). Spearman correlations were carried out to explore the associations between the intake of food groups, foods, dietary compounds, and the fecal NOC concentration. Heatmaps were generated using “corrplot” R package. According to WCRF, a cutoff intake value of 50 g of processed meat per day was selected to analyze differences in fecal NOC concentrations.^1^ The relationship between fecal NOC concentrations and fecal mutagenicity was evaluated by performing simple linear regressions.

## Results

### General Characteristics of the Sample Population

The general characteristics of the human study sample according to postcolonoscopy histopathological diagnosis and nutritional assessment are presented in Table 1. The general sample is mostly composed of women (58%), the age average was 61 years old, with a mean BMI of 26.25 kg/m^2^, which indicates overweight; most of the volunteers were nonsmokers (56%). Out of the total sample, 31% were NP in comparison with 22% in the HP group, 39% displaying CA, and 8% diagnosed with AC. The intake of ethanol was significantly higher in the CA group in comparison with the NP group (16.23 g/day vs 2.93 g/day). No statistically significant differences were found for the rest of the variables analyzed between NP and HP, CA, or AC groups.

**Table 1:** General Description of the Study Sample According to Histopathological Classification^a^

		**WS** (*n* = 36)	**NP** (*n* = 11)	**HP** (*n* = 8)	**CA** (*n* = 14)	**AC** (*n* = 3)
gender	male	15 (41.7%)	3 (27.3%)	3 (37.5%)	6 (42.9%)	3 (100.0%)
	female	21 (58.3%)	8 (72.7%)	5 (62.5%)	8 (57.1%)	0 (0.0%)
age (years)		61 ± 7	59 ± 9	59 ± 7	62 ± 6	64 ± 3
weight (kg)		73.51 ± 14.49	71.14 ± 15.93	74.63 ± 13.62	74.57 ± 16.23	74.33 ± 1.15
height (cm)		166.74 ± 9.79	165.18 ± 12.37	169.25 ± 7.23	164.75 ± 9.24	175.00 ± 2.65
BMI (kg/m^2^)		26.25 ± 3.95	25.66 ± 4.04	25.97 ± 4.21	27.30 ± 4.18	24.28 ± 0.37
energy intake (kcal/day)		1993.30 ± 812.16	1830.12 ± 779.93	1979.17 ± 1005.41	2103.66 ± 837.09	2114.34 ± 393.01
ethanol (g/day)		11.40 ± 20.38	2.93 ± 5.69	17.49 ± 30.16	16.23 ± 21.79 *	3.68 ± 6.38
smoking habit	current	4 (11.1%)	0 (0.0%)	2 (25.0%)	1 (7.1%)	1 (33.3%)
	never	20 (55.6%)	7 (63.6%)	5 (62.5%)	7 (50.0%)	1 (33.3%)
	former	12 (33.3%)	4 (31.3%)	1 (12.5%)	6 (42.9%)	1 (33.3%)
sleeping (h/day)		7.11 ± 1.05	7.10 ± 0.74	7.25 ± 1.28	7.07 ± 1.21	7.00 ± 1.00
physical activity (min/day)		57.29 ± 25.62	59.32 ± 21.33	64.69 ± 23.35	45.54 ± 27.35	85.00 ± 8.66
depositions (times/week)		6.85 ± 2.26	6.91 ± 2.19	6.13 ± 2.74	7.04 ± 2.29	7.67 ± 1.44

a Only individuals with information on fecal NOC concentration and diet are included in the table. Values are presented as mean ± SD for continuous variables or number (%) for categorical ones. (*) Statistically significant differences compared to the NP group (*p* < 0.05) were found with the Mann–Whitney *U* test. NOCs, N-nitroso compounds; BMI, body mass index; WS, whole sample; NP, nonpathological controls; HP, hyperplastic polyps; CA, conventional adenomas; AC, adenocarcinomas.

### Dietary Sources of Nitrates, Nitrites, and Exogenous NOCs (NAs) and Their Association with Fecal NOCs

**Figure 1:**
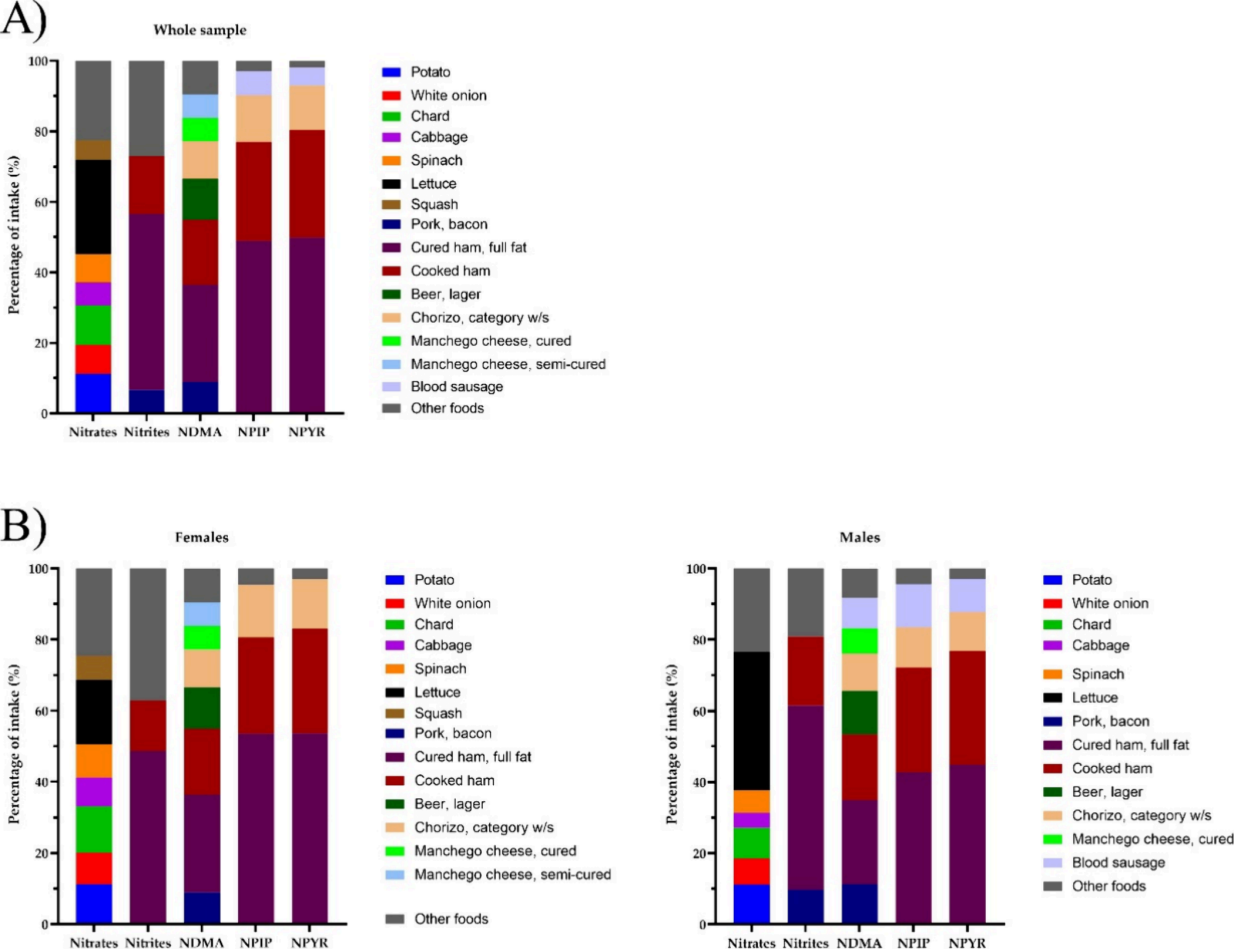
Major dietary sources of nitrates, nitrites, and NAs in (A) the sample population or (B) according to female or male gender. NAs, nitrosamines; NDMA, N-nitrosodimethylamine; NPIP, N-nitrosopiperidine; NPYR, N-nitrosopyrrolidine; W/s, without specifying.

The major foods contributing to the intake of nitrates, nitrites, and the NAs NDMA, NPIP, and NPYR in the total sample are shown in Figure 1A. While the intake of nitrates in the sample is explained by the consumption of roots and vegetables (mainly 27% lettuce, 11% potato, 11% chard, 8% white onion, 8% spinach, 6% cabbage, and 5% squash), nitrites and NAs mainly derive from the consumption of processed meats (such as bacon, ham, or chorizo). Eggs and some plant foods such as potatoes, spinach, onion, and cucumber also contributed as a whole to 14% of the dietary intake of nitrite, although they were represented all together in the category “other foods” in Figure 1 A as their individual contribution was lower than 5%. Furthermore, 12% of the intake of NDMA is provided by beer. Differences in the dietary sources of the compounds under study were also analyzed by gender, and results are presented in Figure 1B. Sources of nitrate were similar for both genders with the exception of squash and cabbage, only identified as a nitrate dietary source in females. Regarding the dietary sources of nitrites, in both genders, the main contributors were cured and cooked ham. Bacon also contributed (>5%) to the intake of this compound, but only in men. In turn, both cured and cooked ham together with chorizo accounted for most of the intake of NDMA, NPIP, and NPYR in both genders as well as blood sausage exclusively in males. No differences in the intake of nitrates, nitrites, and the different NAs according to histopathology groups were detected (Table 2). A Spearman correlation analysis was conducted to further explore the association between the intake of the major dietary sources of nitrates, nitrites, and NAs and the fecal concentration of NOCs (Table 3). From the assessed foods, chard and spinach were negatively correlated with the heme NOCs, contrary to other foods, including potato, bacon, cured ham, chorizo, and blood sausage that were positively correlated with the fecal total NOCs and with heme NOCs. A stepwise regression analysis was performed to determine which of these food sources correlating with fecal total and heme NOC concentrations were predictors of these variables. “Chorizo, category w/s” was revealed as one of the food sources predicting the levels of fecal total and heme NOCs in this sample (Table 4).

**Table 2:** Dietary Intakes of Nitrates, Nitrites, and NAs According to Histopathology Groups^a^

	**WS** (*n* = 36)	**NP** (*n* = 11)	**HP** (*n* = 8)	**CA** (*n* = 14)	**AC** (*n* = 3)
nitrates (mg/day)	127.02 ± 117.03	171.86 ± 166.81	88.3 ± 47.57	112.04 ± 101.29	135.8 ± 84.88
nitrites (mg/day)	3.13 ± 2.32	2.91 ± 1.75	3.02 ± 3.05	3.57 ± 2.58	2.23 ± 0.44
NDMA (μg/day)	0.20 ± 0.17	0.14 ± 0.1	0.23 ± 0.25	0.23 ± 0.17	0.17 ± 0.06
NPIP (μg/day)	0.09 ± 0.07	0.08 ± 0.05	0.09 ± 0.09	0.10 ± 0.07	0.06 ± 0.02
NPYR (μg/day)	0.14 ± 0.10	0.13 ± 0.09	0.13 ± 0.14	0.16 ± 0.11	0.10 ± 0.03

a Values are presented as mean ± SD. No statistically significant differences were found (*p* < 0.05) with the Mann–Whitney *U* test. NAs, nitrosamines; WS, whole sample; NP, nonpathological controls; HP, hyperplastic polyps; CA, conventional adenomas; AC, adenocarcinomas; NDMA, N-nitrosodimethylamine; NPIP, N-nitrosopiperidine; NPYR, N-nitrosopyrrolidine.

**Table 3:** Spearman Correlations between the Intake of the Major Food Sources of Nitrates, Nitrites, and NAs and the Concentration of Fecal NOCs in the Sample Population^a^

**food**	**intake (g/day)**	nitrate (mg/100 g food)	nitrite (mg/100 g food)	NDMA (μg/100 g food)	NPIP (μg/100 g food)	NPYR (μg/100 g food)	**compound**	**Rho (Spearman)**	*p***value**
potato	52.89 ± 30.41	16.8	0.11	0	0	0	Total NOCs	0.431	0.009
							Heme NOCs	0.437	0.008
chard	14.99 ± 42.45	203.0	0.13	0	0	0	Total NOCs	–0.274	0.106
							Heme NOCs	–0.354	0.034
spinach	9.46 ± 17.62	163.0	0.77	0	0	0	Total NOCs	–0.247	0.146
							Heme NOCs	–0.358	0.032
pork, bacon	2.95 ± 6.52	3.2	10.1	1.01	0	0	Total NOCs	0.524	0.001
							Heme NOCs	0.517	0.001
cured ham, full fat	26.19 ± 25.43	2.9	7.20	0.20	0.18	0.29	Total NOCs	0.364	0.029
							Heme NOCs	0.392	0.018
chorizo, category w/s	11.99 ± 17.18	0	0	0.20	0.08	0.12	Total NOCs	0.468	0.004
							Heme NOCs	0.491	0.002
blood sausage	3.91 ± 9.42	0	0	0.35	0.20	0.21	Total NOCs	0.433	0.008
							Heme NOCs	0.419	0.011

a Intake values are presented as mean ± SD. Nitrate, nitrite, NDMA, NPIP, and NPYR concentration values in foods were obtained from EPIC and other authors' data.^17,20−22^ Only dietary sources showing statistically significant Spearman correlation *p*-value <0.05 in at least one fecal NOC variable are shown. NAs, nitrosamines; NOCs, N-nitroso compounds; W/s, without specifying.

**Table 4:** Results Obtained from Stepwise Regression Analyses Identifying Food Sources of Nitrates, Nitrites, and NAs Predictors of Fecal NOCs Concentration^a^

**dependent variable**	**independent variables**	***R***^**2**^	**β**	*p***value**	included*
total NOCs	potato		0.317	0.052	No
	pork, bacon		–0.201	0.205	No
	cured ham, full fat		–0.250	0.165	No
	chorizo, category w/s	0.152	0.420	0.011	Yes
	blood sausage		0.192	0.254	No
heme NOCs	potato		0.273	0.095	No
	chard		–0.100	0.539	No
	spinach		–0.205	0.205	No
	pork, bacon		–0.227	0.149	No
	cured ham, full fat		0.271	0.130	No
	chorizo, category w/s	0.156	0.424	0.010	Yes
	blood sausage		0.167	0.319	No

a Only the variables with *p* < 0.05 in previous correlation analyses are included in the model. * Independent variables showing *p* < 0.05 are finally included in the stepwise regression. NAs, nitrosamines; NOCs, N-nitroso compounds; *R*^2^, coefficient of multiple determination; β, standardized regression coefficient.

### Fecal NOCs (Total and Heme NOCs) Concentrations According to Histopathology Groups

**Figure 2:**
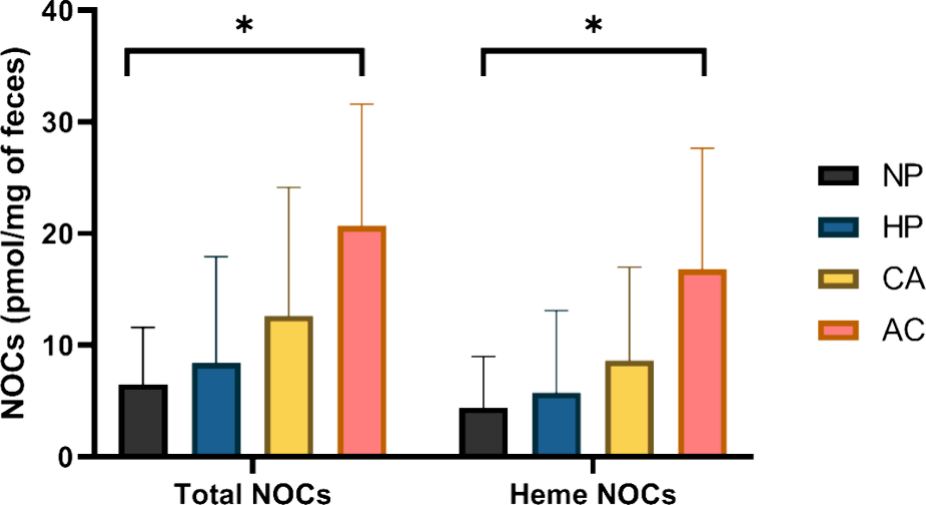
Differences in fecal NOCs concentration according to histopathology groups. Bars represent the mean concentration and vertical lines the standard deviation within each histopathology group. (*) Statistically significant differences were obtained between histopathology groups (Mann–Whitney *U* test, *p* < 0.05). NOCs, N-nitroso compounds; NP, nonpathological controls; HP, hyperplastic polyps; CA, conventional adenomas; AC, adenocarcinoma.

The differences in fecal NOC concentrations according to the histopathology groups are shown in Figure 2. Our data showed an increase in the fecal concentration of total NOCs and heme NOCs as the grade of intestinal mucosa lesion increases from NP to AC, obtaining statistically significant differences between these two extreme groups (NP and AC) for total NOCs (6.50 vs 20.69 pmol/mg of feces, *p* = 0.006) and heme NOCs (4.40 vs 16.80, *p* = 0.017). Although this trend was maintained in each histopathology group, the differences were not statistically significant.

**Figure 3:**
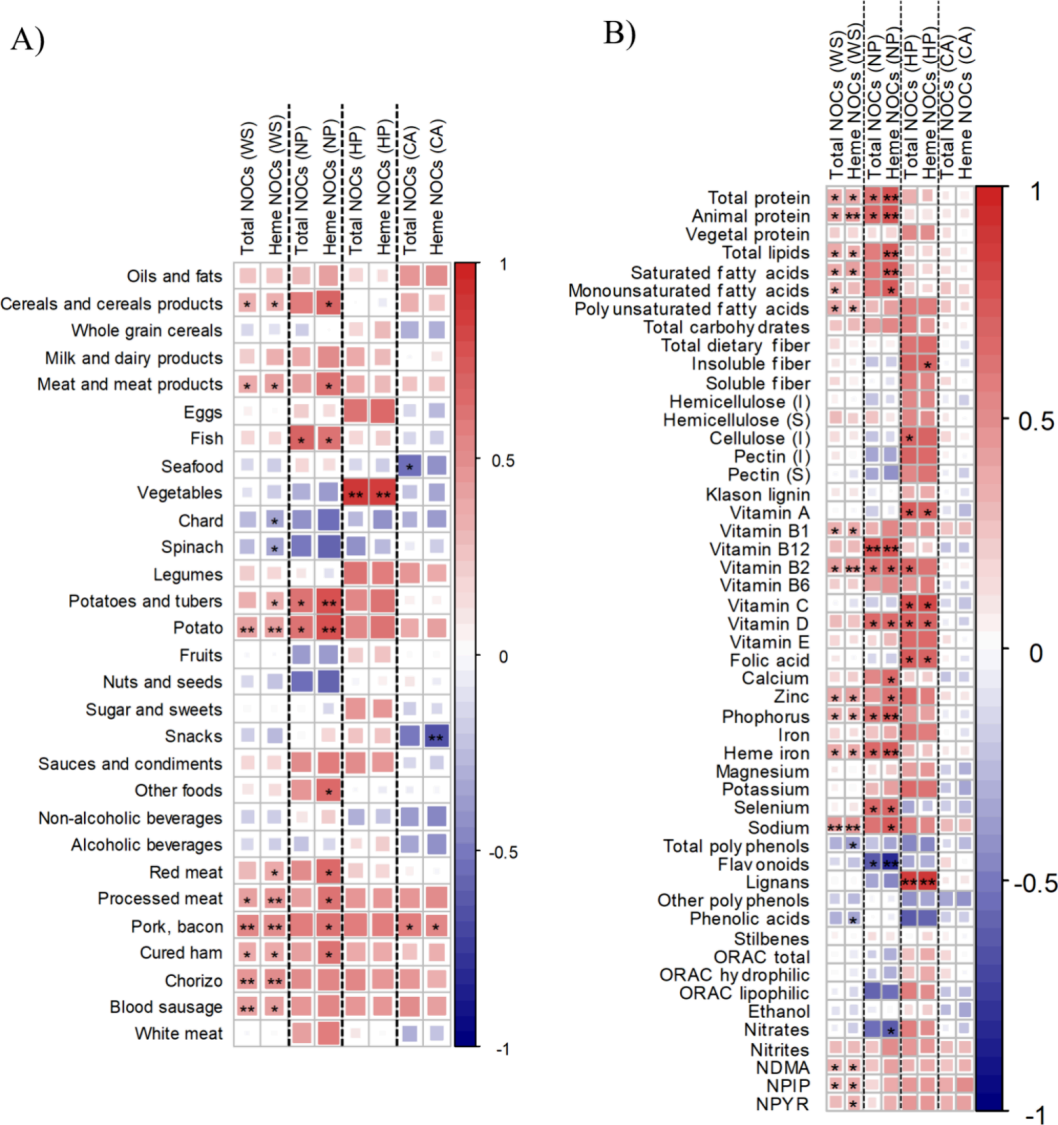
Heatmaps defined by Spearman correlations according to histopathology groups between fecal NOCs and (A) food groups and foods or (B) dietary compounds. Blue and red colors represent negative and positive associations, respectively. The color intensity is proportional to the degree of association. (*) *p* < 0.05 (**) *p* < 0.01. NOCs, N-nitroso compounds; WS, whole sample; NP, nonpathological controls; HP, hyperplastic polyps; CA, conventional adenomas; AC group was not included in the analysis as a result of the limited sample size (*n* = 3); I, insoluble; S, soluble; ORAC, oxygen radical absorbance capacity; NDMA, N-nitrosodimethylamine; NPIP, N-nitrosopiperidine; NPYR, N-nitrosopyrrolidine.

Statistically significant higher fecal NOC concentrations were also observed in volunteers consuming >50 g/day of processed meat, a food group that included different foods, such as bacon, sausages, and cured ham and fermented meats, such as chorizo and blood sausage, in comparison with volunteers consuming <50 g/day, with total NOC concentrations of 12.78 and 7.39 pmol/mg of feces, respectively (*p* = 0.044), and heme NOC concentrations of 9.62 and 4.58, respectively (*p* = 0.017). Focusing on processed meat, a closer examination of associations between fecal NOC concentrations and dietary intake revealed intergroup differences (Figure 3). The whole sample presents positive associations between the intake of processed meats, red meat, cereal products, and potatoes with heme NOC concentrations. The same correlations for the whole sample appeared at higher intensity in the NP as compared with the HP group. Specifically, the NP group displayed a significant positive association of fish consumption with total NOCs and heme NOCs, whereas the same was true for vegetables in the HP group (Figure 3A). In contrast, negative correlations of seafood and snacks with fecal NOCs were observed in the CA group; this group maintained the same positive association found in the other groups for the intake of processed meat and fecal NOCs although these only reached statistical significance for the intake of pork bacon. In Figure 3B, the associations found between dietary compounds and fecal NOCs are depicted. Although the whole sample presented negative correlations for the intake of phenolic acids and total polyphenols with fecal heme NOC concentration, the dietary NDMA, NPIP, or NPYR were positively correlated with total and heme fecal NOCs. Regarding the different histopathological groups, inverse associations were found for the NP group between the intake of nitrates and flavonoids with fecal heme NOC concentrations, whereas positive associations were found between the intake of total protein and micronutrients, such as vitamin B12, vitamin D, phosphorus, and selenium with fecal total and heme NOCs. No significant associations were found in the case of the CA group. Heme iron derived from meat, fish, and eggs intake was positively correlated with total and heme fecal NOC concentrations in the whole sample (*r* = 0.364 and *r* = 0.374, respectively) and NP group (*r* = 0.645 and *r* = 0.745, respectively) but not in HP and CA groups.

### Relationships among Fecal NOCs and Fecal Mutagenicity

**Figure 4:**
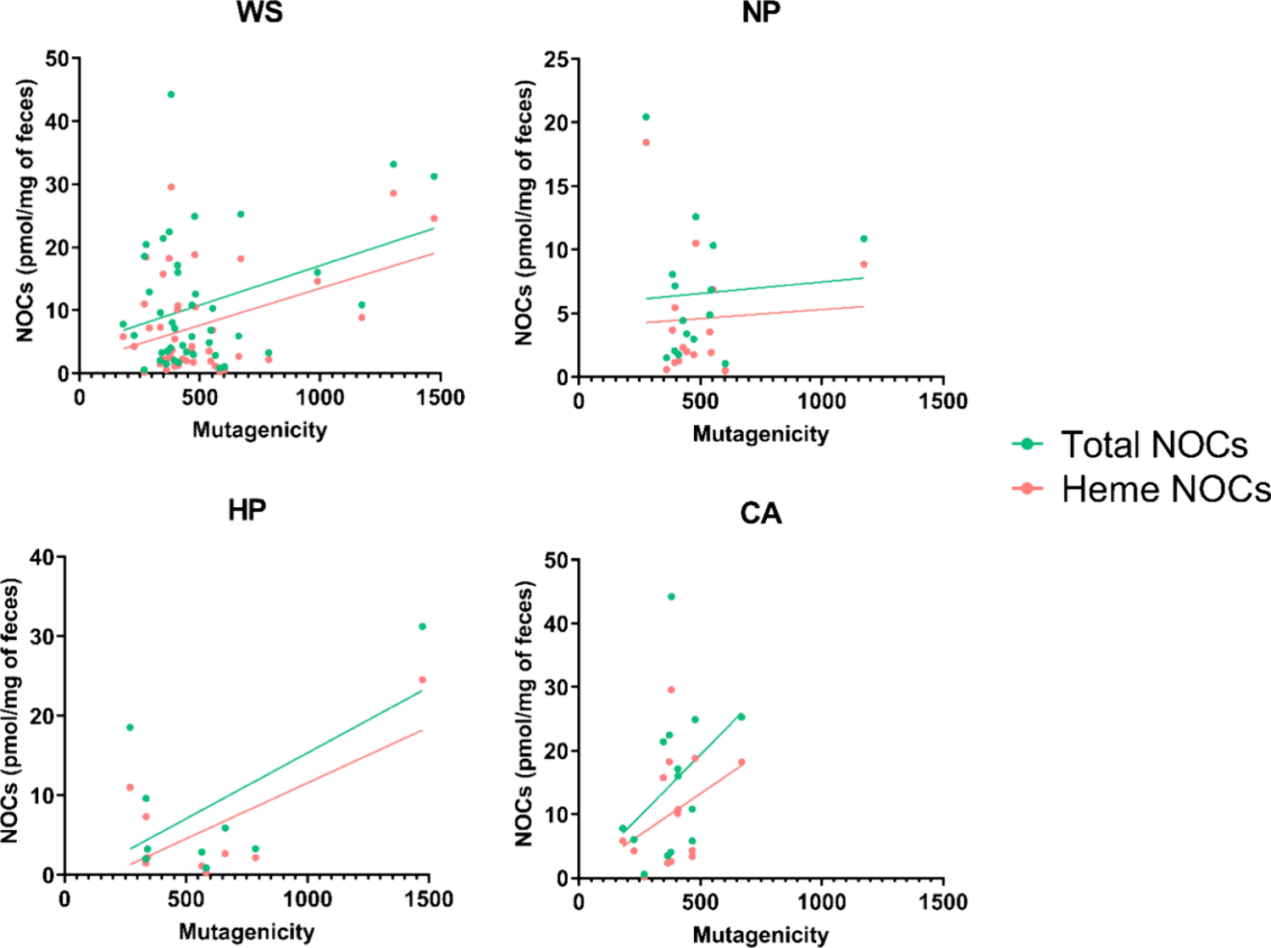
Linear regressions of fecal NOCs depend on the fecal mutagenicity values and according to histopathology groups. NOCs, N-nitroso compounds; WS, whole sample; NP, nonpathological controls; HP, hyperplastic polyps; CA, conventional adenomas; AC, adenocarcinomas.

The concentration of fecal NOCs was evaluated according to the fecal mutagenicity group (Table 5). No statistically significant differences were obtained in the concentration of these compounds as a function of mutagenicity levels, although higher total and heme NOCs were observed in the high mutagenicity group. The relationship was further examined by linear regression analysis (Figure 4). When the whole sample was considered, the fecal concentrations of total NOCs (*R*^2^ = 0.117, *p* < 0.05) and heme NOCs (*R*^2^ = 0.167, *p* < 0.05) increased as fecal mutagenicity increased. The NP group presented the slightest positive association between fecal NOCs and mutagenicity in comparison with the rest of the groups. In the case of the HP group, a significant association between fecal heme NOCs and mutagenicity was found (*R*^2^ = 0.453, *p* < 0.05).

**Table 5:** Fecal NOCs Concentrations According to Fecal Mutagenicity Groups^a^

(pmol/mg of feces)	**low mutagenicity** (0–300)(*n* = 6)	**medium mutagenicity** (>300–600)(*n* = 27)	**high mutagenicity (>600)** (*n* = 8)
total NOCs	11.05 ± 7.76	9.44 ± 9.76	15.85 ± 12.68
heme NOCs	7.81 ± 6.29	6.27 ± 7.78	12.52 ± 10.70

a Values are presented as mean ± SD. No statistically significant differences were found (*p* < 0.05) with the Mann–Whitney test. NOCs, N-nitroso compounds.

## Discussion

**Figure 5:**
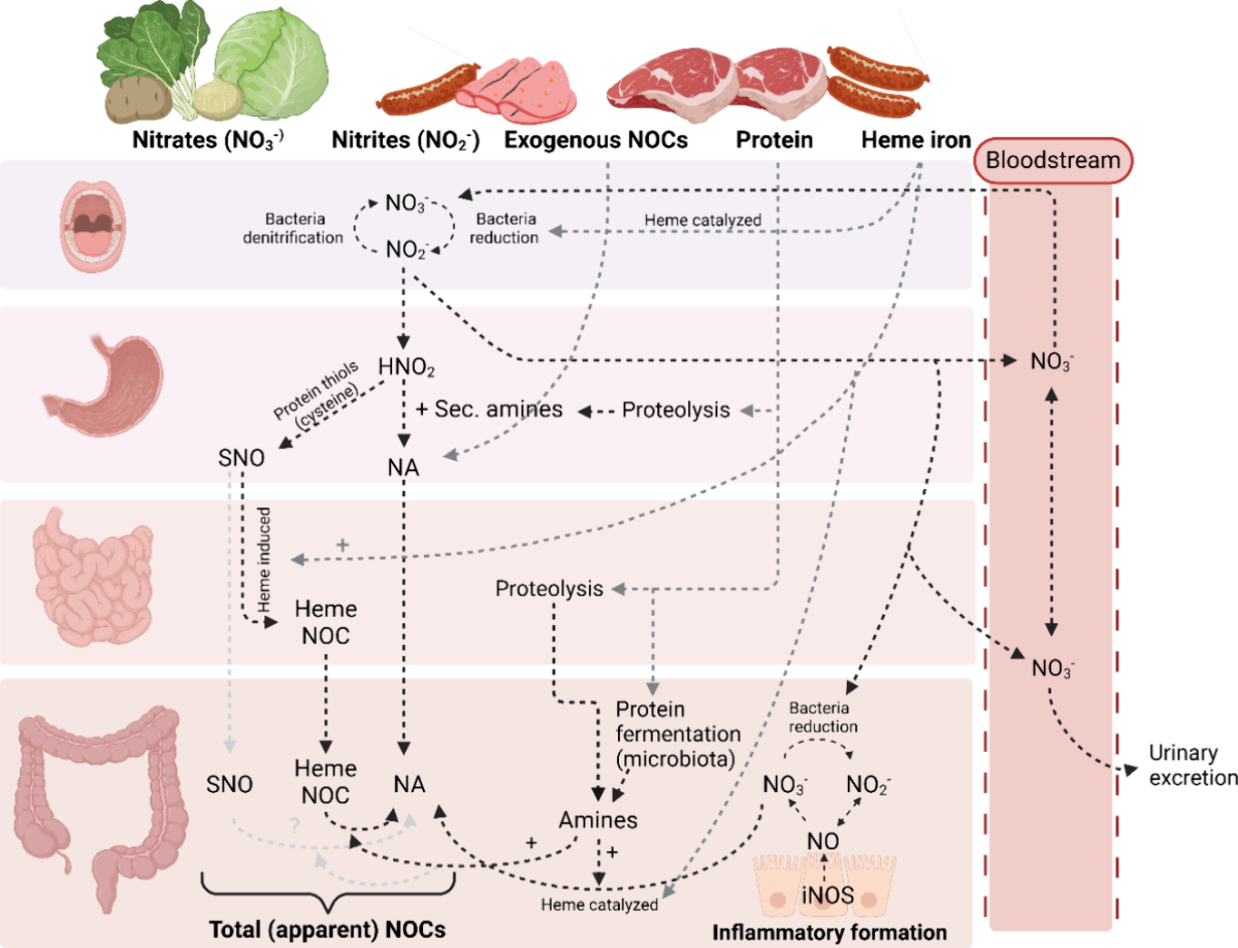
Schematic representation of the mechanisms and dietary factors affecting endogenous NOCs formation and excretion to the stool. Gray arrows indicate dietary sources of precursors and inducers of NOCs formation and black arrows indicate endogenous NOCs formation. NOCs, N-nitroso compounds; NO_3_^–^, nitrate ion; NO_2_-, nitrite ion; HNO_2_, nitrous acid; Sec, secondary; NAs, nitrosamines; SNOs, S-nitrosothiols; Heme NOCs, nitrosyl heme; NO, nitrogen monoxide; iNOS, inducible NO synthase.

To the best of our knowledge, this is the first study investigating the contribution of diet and its components, with special emphasis on NAs derived from food processing and on endogenous NOC formation in humans in the context of CRC. In this work, greater concentrations of fecal total NOCs and heme NOCs were found as the severity of colonic mucosa damage increased. Among the proposed mechanisms to explain the association between CRC risk and higher red and processed meat consumption is the endogenous formation of possible carcinogenic NOCs through the gastrointestinal tract.^14,15,31,32^ The fecal concentration of NOCs depends on many factors such as the intake of exogenous NOCs, nitrate, and nitrites that could act as nitrosating agents, amines, and amides that could be transformed into secondary amines, heme iron from food and the host microbiota (Figure 5).^13,33,34^

Approximately 7% of dietary nitrates can be reduced to nitrite by bacterial nitrate reductase in the oral cavity, for which the heme group acts as an electron donor favoring the catalytic formation of nitrite.^35^ Once in the stomach, nitrite is transformed to nitrous acid due to the low pH conditions and may lead to the formation of NAs and SNOs after reaction with secondary amines and thiol groups obtained from proteolysis of protein food.^13,36^ SNOs facilitate NO release and the nitrosylation of heme iron from meat sources in the small intestine.^37^ Nitrosating agents formed from NO produced by inducible NO synthase (iNOS) in colonocytes together with nitrate, nitrite, heme NOCs, SNOs, and intestinal microbiota could contribute to the further formation of endogenous NOCs in this location.^13,38,39^ Many NAs are carcinogenic compounds, causing damage through DNA alteration.^40^ In addition, the heme group can increase the activity of nitrosating agents and can contribute to DNA damage by increasing lipid peroxidation and generation of cytotoxic and genotoxic aldehydes.^41−43^

Similar to what occurs in most Westernized societies, the average consumption of red (58.27 g/day) and processed meat (64.83 g/day) in the study sample was above the Spanish recommendations for a maximum of 500 g per week (approximately 71 g/day of combined red and processed meat).^44^ In addition, the mean intake of processed meat in the sample of the study was above the risk dose for CRC (50 g/day).^1^ Moreover, volunteers showing processed meat intakes >50 g/day presented higher fecal concentrations of total and heme NOCs. Our findings pointed to processed meats rich in nitrites and NAs, such as bacon, full fat cured ham, chorizo (a Spanish cured sausage), and blood sausage, as the main dietary sources of NOCs associated with fecal total NOCs and heme NOCs. In our results, beer appeared as a dietary source of NDMA based on a reference from an EPIC study that dated back to 1988.^45^ Procedures and standards in the food industry have improved notably in the last decades, which probably has contributed to reduce the originally reported concentration of NDMA in fermented beverages. Interestingly, potato intake was found to be correlated with fecal NOCs, probably because they are often consumed with meat, suggesting a confounding effect as previously reported by other authors.^46^ Among all food sources studied in this work, chorizo was a predictor of the levels of total fecal NOCs in the sample studied. Although the result was statistically significant, the low *R*^2^ value obtained in the model suggested that other factors not considered in this work, such as the intestinal microbiome, could be playing an important role in NOC formation. It is also noteworthy that the intake of phenolic acids and total polyphenols was negatively correlated with fecal heme NOCs, suggesting an inhibitory effect of these plant-derived bioactives on endogenous NOC formation.

Case-control studies are often influenced by selection, recall, and reporting biases when dietary intake assessment is done after diagnosis. However, since intestinal polyps are often asymptomatic, it is less likely that patients have altered their dietary intake before the visit to the hospital. The methodology used for registration of the dietary intake and the further databases employed for conversion into different dietary compounds with carcinogenic and bioactive effects have allowed us to obtain a high degree of detail on dietary intake information. However, differences in the intake of nitrates, nitrites, NDMA, NPIP, and NPYR among the few studies available in the literature are to be expected due to the different methods of dietary analysis used in each case. In this regard, Holtrop et al. determined that diet composition was associated with the endogenous formation of NOCs in a sample population of obese men. For that purpose, they used the McCance and Widdowsons’ tables with semiquantitative data from different food categories instead of using a detailed estimation of the intake of each individual food item.^47^ The use of dietary history questionnaires reported lower mean intakes of nitrites (0.99 vs 3.13 mg/day in our study) and NDMA (0.114 μg/day vs 0.198 μg/day in our study) in a Spanish population as compared to the intakes obtained nearly 30 years ago in a Finnish sample population (5.30 mg/day of nitrite and 0.05 μg/day of NDMA).^48,49^ Regarding the intake of NPYR and NPIP, previous studies are scarce. In our sample, we detected an intake of 0.138 and 0.088 μg/day of NPYR and NPIP, respectively. Dietary estimations from German nutritional surveys set NPYR and NPIP exposure to 0.011 and 0.015 μg/day, respectively, but this study did not detail each meat consumption level, which could have helped to better understand the differences between the results.^50^

One of the main objectives of this work was to determine whether the estimation of dietary nitrates, nitrites, and NAs could be representative and could display a meaningful relationship with the concentrations of NOCs excreted. The whole sample presented nonsignificant correlations between the intake of nitrates and nitrites, respectively, and fecal NOC concentrations. However, the intake of NDMA, NPIP, NPYR, and heme iron from meats, eggs, and fish was significantly and positively correlated with fecal total and heme NOCs, in concordance with previous studies that pointed to their direct association with NOC formation.^13^ Although the statistical significance of these associations was not maintained within the groups established according to the damage of the colorectal mucosa, probably due to the limited sample size and dispersion of the data, similar trends were noted in the case of the food dietary sources of these compounds. Specifically, heme NOCs were positively and significantly associated with the intake of bacon and cured ham in the NP group and with bacon in the CA group, which also showed increased consumption of ethanol. Moreover, we found that vitamin C, an antioxidant molecule previously described as an inhibitor of NOC formation, was positively correlated with fecal total and heme NOCs in the HP group, probably because of the strong correlation of vegetable consumption with fecal NOCs in the same group.^33^ In this work, the vegetables red pepper and green pepper, followed by tomato, were the major foods contributing to vitamin C intake in our sample.

In a previous study by other authors, high NOC concentrations were associated with longer transit time and lower fecal weight, suggesting more efficient microbiota mediated formation and accumulation of these compounds in feces.^51^ In contrast, a higher intake of dietary fiber can increase stool volume and shorten transit time, leading to lower NOC concentrations and decreasing their interactions with the colon mucosa. In this study, it was not possible to calculate daily excretion of NOCs. Therefore, the concentrations reported here could be influenced by confounding factors, such as fecal volume and transit time.

Although with these data we cannot establish causality between the intake of red and processed meats, nitrates, nitrites, and NAs derived from food processing with the endogenous formation of NOCs, it seems clear from our results that these factors are associated. Therefore, the next question is whether this increased excretion of NOCs is associated with increased fecal toxicity. Some previous work addressing this issue had found an increase in fecal water genotoxicity after red meat consumption during an intervention study although nonsignificant increments of NOC fecal levels were observed.^52^ In our study, the group of high fecal mutagenicity showed the highest total and heme fecal NOC concentrations, and according to the histopathology group, strong associations between fecal heme NOCs and fecal mutagenicity were notable only in the case of the HP group.

In the present work, no differences were found in the intake of nitrites and dietary NOCs between the histopathology groups, but we observed that these compounds were mainly derived from the consumption of processed meats and were positively correlated with fecal total and heme NOC concentrations. Increased fecal NOC concentrations were noted among individuals consuming higher amounts of processed meat than recommended by regulatory agencies as well as in association with the increase in the severity of colonic mucosal damage from NP to AC, and in samples with higher fecal mutagenicity. The study of the associations among dietary components and endogenous NOCs considering the different intestinal environments may help to understand their impact on colonic mucosal damage and the progression of certain diseases, such as CRC.
